# Isolation and analysis of rereplicated DNA by Rerep-Seq

**DOI:** 10.1093/nar/gkaa197

**Published:** 2020-04-02

**Authors:** Johannes Menzel, Philip Tatman, Joshua C Black

**Affiliations:** 1 University of Colorado Anschutz Medical Campus, Department of Pharmacology, 12800 E 19th Ave, Aurora, CO 80045, USA; 2 University of Colorado Anschutz Medical Campus, Molecular Biology Graduate Program, 12800 E 19th Ave, Aurora, CO 80045, USA; 3 University of Colorado Anschutz Medical Campus, Medical Scientist Training Program, 12800 E 19th Ave, Aurora, CO 80045, USA

## Abstract

Changes in gene copy number contribute to genomic instability, the onset and progression of cancer, developmental abnormalities and adaptive potential. The origins of gene amplifications have remained elusive; however, DNA rereplication has been implicated as a source of gene amplifications. The inability to determine which sequences are rereplicated and under what conditions have made it difficult to determine the validity of the proposed models. Here we present Rerep-Seq, a technique that selectively enriches for rereplicated DNA in preparation for analysis by DNA sequencing that can be applied to any species. We validated Rerep-Seq by simulating DNA rereplication in yeast and human cells. Using Rerep-Seq, we demonstrate that rereplication induced in *Saccharomyces cerevisiae* by deregulated origin licensing is non-random and defined by broad domains that span multiple replication origins and topological boundaries.

## INTRODUCTION

Eukaryotic cells maintain tight control over DNA replication to guarantee that each daughter cell receives only one copy of the genetic material. Rereplication, the initiation of DNA replication more than once per cell cycle, is critical in development of worms and flies and has been proposed as an important source of gene amplifications and aneuploidy, which are critical parameters that contribute to tumorigenesis and tumor progression (1–4). Deregulation of cell cycle dependent control of DNA replication, through pharmacological disruption or genetic perturbations, can result in uncontrolled rereplication (5–10). Moreover, induced rereplication is a potent inducer of gene amplification (11). Consistent with these observations, DNA replication and licensing factors are overexpressed or amplified in numerous tumors (12,13). However, an understanding of the extent of DNA rereplication in development, disease, and cancer has remained elusive due to the lack of a generally applicable methodology to identify rereplicated DNA. Current strategies for evaluating DNA rereplication study individual loci using DNA fluorescent *insitu* hybridization, total DNA content of cells by flow cytometry or analysis by CsCl gradient centrifugation for rereplicated DNA. Of these methods, only CsCl gradient centrifugation has the potential to reveal global sequence-specific information, but requires large amounts of input DNA (as much as 100 μg), preparation time and optimization for individual species due to varying GC content of DNA. These methods have been unable to answer key questions about DNA rereplication: is DNA rereplication stochastic; what specifies regions for rereplication; how does rereplication contribute to disease etiology? To address these questions, we must be able to determine which genomic regions rereplicate under which specific conditions.

To overcome this challenge, we have developed Rerep-Seq, a technique that selectively fragments and enriches rereplicated DNA, from high molecular weight genomic DNA, for use in contemporary sequencing methods. Rerep-Seq uses small amounts of DNA (1–5 μg), and can be applied to any species or sample capable of incorporating BrdU with a rapid experimental timeline. These features will permit application of Rerep-Seq on diverse samples including model organisms and cultured tissue biopsies. Here we validate the versatility of Rerep-Seq to detect and identify rereplicated DNA from yeast and human models of rereplication. At last, using Rerep-Seq we demonstrate that rereplication caused by inducible bypass of the licensing machinery in budding yeast results in non-random rereplication of broad domains that span multiple origins of replication and cross topological boundaries. Our results describe a new technology, which can be used to address the extent and specificity of DNA rereplication in development and disease.

## MATERIALS AND METHODS

### Yeast strains, plasmids, media and growth conditions

Yeast strains used in this study are listed in Table 1 and were grown according to standard procedures in YPD (yeast extract, peptone and 2% dextrose) at 30°C for all experiments unless otherwise stated (14). To accommodate *Saccharomyces cerevisiae*’s lack of the thymidine salvage pathway, yeast strains in this paper are all stably transformed with an NheI linearized p403–BrdU–Inc HIS3, a reconstituted thymidine salvage pathway cassette, consisting of Herpes simplex virus thymidine kinase (HSV–TK) and human equilibrative nucleoside transporter (hENT1), that enables efficient cellular uptake and incorporation of the thymidine analogue BrdU into DNA (15). Inserts were confirmed by spots assays for sensitivity to 75 ug/ml FUDR (5-fluorodeoxyuridine) and susceptibility to BrdU uptake and genomic fragmentation via Rerep-digest.

**Table 1.** *Saccharomyces cerevisiae* strains used in this study

Yeast Strain	Genotype	Background/Reference
YJB1	MATa ade2-1 his3-11,15 leu2-3,112 trp1-1 ura3-1 can1-100 bar1::hisG ars608Δ::HIS3 ars609Δ::TRP1 ars305::TRP1 GPD-HSV-TK ADH1-hENT1 BrdU-Inc	W303a RSY1296/ YZy50 Zhong *et al.* 2013 (ref 38)
YJB16	MATa ade2-1 ura3-1 his3-11,15 trp1-1 leu2-3,112 can1-100 Δbar1::hisG CDC45 H22Y ura3-1::GAL-sid2-11D-MHT (URA3) leu2-3,112::GAL-DBF4-MHT (LEU2)	W303a/ YST575 Tanaka *et al.* (2007) (ref 29)
YJB18	MATa ade2-1 ura3-1 his3-11,15:: HIS3 GPD-HSV-TK ADH1-hENT1 BrdU-Inc trp1-1 leu2-3,112 can1-100 Δbar1::hisG CDC45 H22Y ura3-1::GAL-sid2-11D-MHT (URA3) leu2-3,112::GAL-DBF4-MHT (LEU2)	W303a/ YJB16 + p403–BrdU–Inc HIS3 (this study)

Black lab yeast strain name, genotype and background/reference.

### Tissue culture, media, growth conditions

MDA-MB-231 cells were maintained in Dulbecco's-modified Eagle's medium (DMEM) with 10% fetal bovine serum, 1% penicillin/streptomycin and L-glutamine at 37°C with 5% CO_2_. 7.5 × 10^5^ cells were seeded on 10 cm plates in 10 ml complete DMEM for culture maintenance.

### DNA extraction

As Rerep-Seq selectively fragments rereplicated DNA and enriches those sequences by size selection, it is imperative that the genomic input DNA is of high molecular weight. For yeast samples: cells were pelleted by centrifugation at 3000 rpm for 3 min and resuspended in SCE (1 M sorbitol, 100 mM sodium citrate, 10 mM ethylenediaminetetraacetic acid (EDTA) pH 8.0); fresh 0.125% (v/v) β-mercaptoethanol and 10 U/ml zymolyase was added and incubated for 30–60 min at 37°C to digest cell walls; human cells or yeast spheroplasts were pelleted by centrifugation at 3000 rpm for 3 min and resuspended in 500 μl RIPA buffer (10 mM Tris–Cl pH 8.0, 1 mM EDTA, 1% Triton X-100, 0.1% sodium deoxycholate, 0.1% sodium dodecyl sulphate (SDS), 140 mM NaCl, 1 mM phenylmethylsulfonyl fluoride (PMSF)) with RNase A, 0.2 mg/ml and incubated at 37°C for 1 h. A total of 25 μl 20% SDS and 10 μl of 20 mg/ml proteinase K was then added and allowed to incubate at 55°C for 2 h. DNA was extracted twice using Phenol:Chloroform:Isoamyl alcohol (25:24:1, v/v) and precipitated using isopropanol, washed in 70% ethanol, then air dried for 5 min. The DNA pellet was resuspended and stored in nuclease free water at −20°C. DNA concentration was determined with Qubit^®^ dsDNA BR Assay Kits, catalog # Q32850.

### Rerep-Seq digestion

In an 8 strip 200 μl polymerase chain reaction (PCR) tube (USA Scientific 1402–4700), 1–5 μg high-molecular weight genomic DNA was mixed with 2.5 μl 10× Hoechst 33258 (0.1 mg/ml) and 2.5 μl 10× NEB Buffer 4 (50 mM Potassium Acetate, 20 mM Tris-acetate, 10 mM Magnesium Acetate, 1 mM DTT pH 7.9 @ 25°C) to a final volume of 24 μl; open tubes placed upright in PCR tube rack, covered with a glass plate (3" × 3" glass plate from VWR Vertical Gel Electrophoresis Systems), exposed to 7.5 min of glass plate filtered (UVA only) from a Stratalinker. This strategy blocked greater than 90% of UVB and UVC according to measurements made with a UV radiometer. Following UVA treatment, samples were digested with 0.5 μl UDG (five units of Uracil-DNA Glycosylase) NEB catalog number M0280S, and 0.5 μl APE1 (10 units of human apurinic/apyrimidinic endonuclease 1) NEB catalog number M0282S for 2 h at 37°C. Digested DNA was repaired with NEB’s FFPE DNA Repair Mix, NEB catalog # M6630L, for 30 min then separated on 0.8% agarose gel for 15 min at 200V. Fragmented DNA ranging from 0.1 to 3 Kb was gel extracted with Wizard^®^ SV Gel and PCR Clean-Up System (Promega A9281) and the purified DNA was resuspended in 50 μl of nuclease free water and stored in low adhesion tubes (USA Scientific 1415–2600) at −20°C for subsequent qPCR and sequencing analysis.

### Quantitative PCR

Real-time or quantitative PCR (qPCR) was carried out for each DNA sample using iTaq Universal SYBR Green Supermix (BioRad 1725121). Gel extracted digested DNA ranged in concentration from 0.1 to 15 ng/μl. Therefore we only assessed concentration by fluorimetry using Qubit^®^ dsDNA HS Assay Kits, catalog # Q32854. Equal volumes of Rerep-Seq digest samples where used in each reaction. Each PCR reaction contained: 0.5 μl Rerep-Seq digest samples, 5.0 μl 2× iTaq Universal SYBR Green Supermix, 0.3 μl mixed forward and reverse primers at 10 μM and 4.2 μl water for a total of 10 μl per reaction well. For each sample and primer pair combination, an iTaq/sample master mix and a water/primer master mix was manually prepared. This enabled measurement of the internal mitochondrial genome control from the same mastermix (and DNA concentration) as the target genomic loci. PCR reactions were run on the CFX384 Touch Real-Time PCR Detection System (BioRad) with software CFX Manager Version 3.1.1517.0823. DNA was amplified with a program consisting of an initial 3 min 95°C denaturation, then 45 cycles of 5 s 95°C denaturation, 30 s 60°C annealing/extension followed by a melting curve analysis from 65°C to 95°C at 0.5°C/cycle. Each of three biological replicates was measured in technical triplicate (three wells of qPCR). For both human and yeast, DNA sequence quantification was normalized to the mitochondrial gene CO×2. The three technical triplicate Cq values for each primer set were averaged for a single Cq value for each biological replicate. This Cq value is converted to 2^-Cq value. The 2^-Cq values of the region of interest is divided by the mitochondrial DNA normalizer (COX2) 2^Cq value of the same sample. All primers were acquired from Sigma-Aldrich. Oligonucleotide sequences used are listed in Table 2. Each primer set was assessed for specificity by melting curve analysis and each exhibited a single peak among replicates.

**Table 2.** Primers used in this study

Species	ID	Forward (5′-3′)	Reverse (5′-3′)	Location	Length
*S. cerevisiae*	ACT1	GGTGTCTTGGTCTACCGACG	TGTGTAAAGCCGGTTTTGCC	chrVI:54 267–54 341	75 bp
*S. cerevisiae*	ARS307	AGCAGTAGCACATGGACACA	ACTTTCTTGTGTGGGCTGCT	chrIII:108 976–109 038	63 bp
*S. cerevisiae*	COX2	TTAAAGTTGATGCTACTCCTGGT	TTTGCATGACCTGTCCCACA	chrM:74 341–74 449	109 bp
*H. sapiens*	ACTb	TCCAAAGGAGACTCAGGTCAG	CGCCCTTTCTCACTGGTTC	chr7:5 529 028–5 529 100	73 bp
*H. sapiens*	Tel16	TTCTCCCTCCCCCTTGATT	AGGGACAAAGAAATGGAAGGA	chr16:46 619 343–46 619 402	60 bp
*H. sapiens*	HCN1	CGTGCTCTTGTGCACTTCAT	CAGCAGCAGGTACAGCAGTC	chr5:45 262 281–45 262 391	111 bp
*H. sapiens*	hCOX2	CCCCACCCTACCACACATTC	GCTTGAAACCAGCTTTGGGG	chrM:7399–7487	89 bp

Names of DNA oligos used for qPCR, sequences and target species and genomic location. Human positions are in hg38 and yeast in sacCer3.

### Sequencing library construction

Sequencing libraries were constructed using the Illumina Nextera DNA Flex Library Prep Kit (catalog number 20018704) following the manufacturers’ protocol and using 14 cycles of amplification for final library construction. Libraries were constructed using dual index barcodes from the Nextera DNA CD Indexes (catalog number 20018707). Pooled libraries were sequenced by Novogene (Novogene Corporation INC Chula Vista CA) to obtain ∼10 million reads per yeast sample and 30 million reads per human sample.

### Yeast simulated DNA rereplication

For the synchronized replication timing experiment, yeast cells were arrested in G1 with α-factor (100 ng/ml) for 2 h, then released into fresh media containing 0.1 mM BrdU to label one DNA strand. To maintain synchronization the culture was arrested with α-factor a second time, 40 min post-release. This second G1 arrested culture was released again into media containing 0.1 mM BrdU and 50 μg/ml pronase. Flow cytometry and DNA samples were collected to monitor S-phase progression. Each of three individual replicates was performed on separate days from a single colony (different for each replicate) grown in 50 ml YPD overnight at 30°C then diluted to an OD^600^ of 0.5 in 100 ml pre-warmed YPD prior to α-factor arrest. Cultures were release by centrifugation at 2000 rpm for 3 min then resuspended into fresh pre-warmed YPD, 200 ml for first release, 500 ml for second release and 50 ml of culture was collected for each timepoint sample.

### Human simulated DNA rereplication

For G2/M arrest, 2.0 × 10^6^ cells were seeded into p15 tissue culture plates and were grown for 48 h, followed by treatment with 30 μM BrdU for 4 h prior to addition of nocodazole to 12.5 ng/ml for an additional 12 h. For release, non-adhering G2/M phase arrested cells were tapped and rinsed off the plate, washed twice with media via centrifugation at 1000 rpm for 3 min and resuspended in 10 ml fresh complete DMEM, then plated on new 10 cm plates in 10 ml media containing 30 μM BrdU. Samples were collected at 0, 10, 15 and 25 h after release for DNA and flow cytometry analysis. Each of three individual replicates was performed on the same MDA-MB-231 cell line on separate weeks, separated by two or three passages of the cells.

### Flow cytometry

For DNA content analysis, 100 μL of yeast culture, OD^600^ of 0.5–1.0 or ∼10^6^ human cells were washed in phosphate-buffered saline (PBS) (NaCl 137 mM, KCl 2.7 mM, Na2HPO4 10 mM, KH2PO4 1.8 mM), fixed by resuspension in ice cold 70% ethanol overnight. Prior to flow analysis, cells were washed once with PBS and then resuspended in PBS. Cells were treated with 0.2 mg/ml RNase A and stained with 10 μg/ml propidium iodide (PI) for 60 min before analysis on a FACScan instrument (BD). Yeast were treated with 20 mg/ml Proteinase K at 55°C for 1 h prior to PI staining as performed for human cells. Data analysis was performed using FlowJo V10.

### CDK bypass rereplication

YJB18 cells (see Table 1) were grown in YP 2% raffinose to an OD^600^ of 0.5, arrested in G1 with α-factor (100 ng/ml) for 2 h. A total of 0.1 mM BrdU was added and the culture was split into two, with 2% galactose added to one for induction of cdc45 H22Y and sld2-11D, while the other was maintained in 2% raffinose. Samples were collected at 0 and 5 h for flow cytometry and DNA extraction. Each individual replicate was performed from a single colony (different for each replicate) on separate days grown in 50 ml YP 2% raffinose overnight at 30°C then diluted to an OD^600^ of 0.5 in 100 ml pre-warmed YP 2% raffinose prior to α-factor arrest.

### Rerep-Seq alignment and normalization

FASTQ files for human samples were aligned to HG38 using Bowtie2. Duplicate reads were removed and bam files were generated using samtools (version 1.7). Bedtools (version 2.26.0) was used to generate a bedGraph and R (version 3.6) was used to RPM normalize and scale the data based on the percentage of reads that aligned to mitochondrial DNA, yielding a normalized bedGraph. Blacklisted regions were removed from these files. We defined a blacklisted region as any region greater than one standard deviation over the average genomic signal in time point 0 from all replicates (these regions are available on our github https://github.com/blacklabUCD/ReRepSeqMethods). The normalized bedGraphs were binned to 1 kb and smoothed using a 100 kb window. The binning algorithm produced identical bins in every sample, allowing samples to be easily averaged. The smoothing window algorithm started and stopped smoothing at half the length of the smoothing window from either end of the chromosome. The samples were merged and averaged using Bedtools (version 2.26.0). Reproducibility of replicates was confirmed using a spearman rank test in R (version 3.6; Supplementary Figure S1).

Yeast FASTQ files were aligned to SacCer3 using Bowtie2. Duplicate reads were removed and bam files were generated using Samtools (version 1.7). BedGraphs were generated using bedtools (version 2.26.0) and R (version 3.6) was used to RPM normalize and mitochondria-scale the data. Blacklisted regions were then removed using a custom blacklist. We defined a blacklisted region as any region greater than one standard deviation above the average genomic signal in all timepoint zero replicates (these regions are available on our GitHub (https://github.com/blacklabUCD/ReRepSeqMethods). We then binned the final bedGraphs using 100 bp regions and smoothed the data to 10 kb, as described above. Reproducibility of replicates was confirmed using a spearman rank test in R (version 3.6; Supplementary Figure S1).

Normalized bedGraphs were imported into R and genomic regions were visualized using the R package Gviz. The ERD (early replicating domain), LRD (late replicating domain), ARS (autonomously replicating sequence) and TADs (Topologically Associating Domains) files displayed in these figures can be found on our GitHub https://github.com/blacklabUCD/ReRepSeqMethods.

### Early replicating domains (ERDs), late replicating domains (LRDs) and autonomously replicating sequence (ARS) analysis

To identify signal enrichment in known replication timing domains in human Rerep-Seq data, we used established replication timing regions derived from MCF7 cells (16). These timing regions were computationally defined using a machine learning algorithm that identified early and late replicating regions (ERD and LRD, respectively), as well as transition zones (TZ) (16). These domains were lifted from hg19 to hg38, using UCSC genome tools. To capture the boundaries of enrichment in ERD and LRD domains, we adjusted the left and right boundary to the midpoint of the neighboring TZ, or chromosome edge. The resulting classification allowed us to capture the spread of replication signal beyond the boundaries of each region, without analyzing the TZs between ERD and LRD regions twice. This data for each timing domain was then rank-ordered by signal intensity and displayed as a heatmap using R (version 3.6). To generate an average profile of these domains, we took the average across all ERDs or LRDs for timepoint and plotted the resulting trace using R (version 3.6).

To visualize yeast timing domain enrichment, we displayed confirmed ARS (17) sequences ±25 kb from the center of the sequence as a heatmap rank-ordered by signal (row sum) using R (version 3.6). We then identified these regions as early or late ARSs using data from published yeast replication timing experiments (18). The published data were presented on a scale from 0 to 2, where 2 was considered replicated and 1 was considered not-replicated. This allowed us to define continuous replicated and non-replicated regions by selecting the upper and lower quartiles of data. We defined early regions as any continuous region in the top quartile of signal intensity, and we defined late regions as any region in the bottom quartile of signal intensity. Using these regions, an average profile was generated and the resulting line was plotted for each timepoint using R (version 3.6).

We used GSEA (version 4.0.3) to quantify the enrichment of ERDs and LRDs in the human and yeast time courses. We created custom early or late ‘gene’ lists from the known early and late replicating ARSs in yeast and ERDs and LRDs in human data. We then created separate ranked lists based on the average signal over all timing domains for human and all confirmed ARS regions in yeast and determined the enrichment of early or LRDs in the list (Supplementary Figures S1B and 2B).

### Code availability

The code used to analyze the data is available in our GitHub https://github.com/blacklabUCD/ReRepSeqMethods. We provide the code freely for all users for whatever purpose. Figures 3, 5, 6 and Supplementary Figures S1–3 all required custom written code to generate. This code is available on our GitHub page. The GitHub page also includes all custom generated bed files for ARS, ERD, LRD, topological domains and blacklisted files used for data analysis.

## RESULTS

### Fundamentals of Rerep-Seq

**Figure 1.**
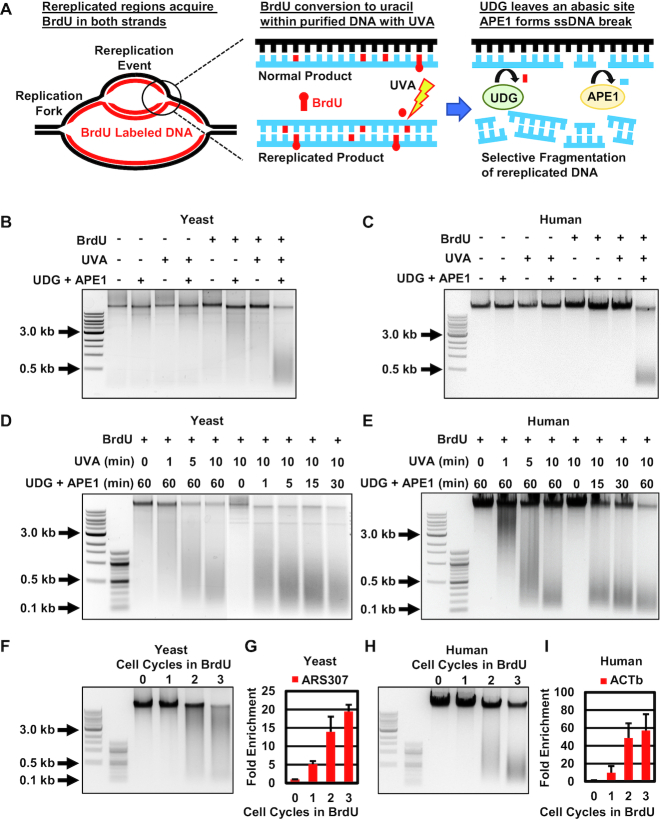
Fundamentals of Rerep-Seq. (**A**) Schematic of selective fragmentation of rereplicated DNA. Replicating DNA incorporates BrdU into newly synthesized strand, rereplicating DNA acquires BrdU in both strands. Rerep-Seq digest: UVA exposure photolyzes incorporated BrdU to produce uracil, followed by nucleotide excision by UDG, to produce an abasic site and generation of single strand breaks through digestion with APE1. Normally replicated DNA generates single stranded breaks and staggered double stranded breaks in rereplicated DNA. (**B** and**C**) Selective fragmentation of yeast (B) and human (C) genomic DNA requires BrdU, UVA and UDG/APE-1 digestion. Genomic DNA from cells labeled with BrdU for zero or two cell cycles (−,+ BrdU) to mimic double labeled rereplicated DNA was treated with the indicated steps of the Rerep-Seq digest procedure. (**D** and**E**) Optimization of fragment size for yeast and human DNA. Genomic DNA exposed to varying doses of UVA followed by treatment with UGD/APE1, and DNA exposed to UVA followed by varying UDG and APE1 digestion time. (**F** and**G**) Selective fragmentation and enrichment of double BrdU labeled yeast DNA. (**H** and**I**) Selective fragmentation and enrichment of double BrdU labeled human DNA. Rerep-digest on genomic DNA treated with BrdU for 1, 2 or 3 cell cycles. DNA fragments were gel extracted and equal volumes were analyzed by qPCR with primers within ARS307 (yeast) and ACTb gene (human). Data represent the average of three biological replicates (*n* = 3), error bars represent the SEM.

Rerep-Seq leverages the semiconservative nature of DNA replication to selectively fragment and enrich rereplicated DNA. Cells are labeled with the thymidine analog BrdU for one cell cycle to allow BrdU incorporation into the newly replicated DNA (Figure 1A; left panel). Replication results in BrdU incorporation into a single strand of DNA, whereas rereplication results in BrdU incorporation into both strands.

Genomic DNA from cells labeled with BrdU is purified and subjected to biochemical processing to induce ssDNA breaks at the sites of BrdU incorporation. DNA is subjected to UVA treatment in the presence of Hoechst 33258 to photolyze the bromine from BrdU leaving deoxyuracil (Figure 1A; middle panel). Deoxyuracil is then removed by treatment with UDG (uracil DNA glycosylase) leaving an abasic site. The abasic site is then converted to a single strand DNA break by deoxyribose excision by APE1 (apurinic/apyrimidinic endodeoxyribonuclease), yielding single DNA nicks in normally replicated DNA, but staggered nicks (i.e. double-stranded DNA breaks) in rereplicated DNA (Figure 1A; right panel). The fragmented, rereplicated DNA can then be isolated by size fractionation and analyzed by quantitative PCR or next-generation sequencing.

### Parameters affecting Rerep-Seq shearing of DNA

The amount of rereplicated DNA fragmentation can be empirically controlled to match the experimenter's preferences (Figure 1B–E). Each step in the Rerep-Seq digest procedure: BrdU labeling, UVA treatment and UDG+APE1 digestion are all required for DNA fragmentation of both yeast and human DNA (Figure 1B and C). Each of these steps can be used to control the extent of fragmentation. In particular, the amount of UVA exposure time is a strong determinant of DNA shearing (Figure 1D and E), which can be refined by altering the amount of time of the UDG and APE1 enzymatic digestion. Empiric testing of these conditions resulted in the use of 30 μM BrdU for human cells and 0.1 mM BrdU for yeast cells with 7.5 min of UVA and 2 h of digestion to generate fragments centered from 300 to 600 bp for optimal library construction using the Illumina Nextera DNA Flex Library Prep Kit (19). Using these conditions, we validated Rerep-Seq fragmentation by labeling yeast and human cells for up to three cell cycles to determine whether Rerep-Seq enriched DNA only in cells labeled for more than two cell cycles with BrdU, thus simulating rereplication (Figure 1F–I). Gel extracted fragmented DNA was analyzed with qPCR (Figure 1G and I). We were able to fragment and significantly enrich DNA only from cells labeled with BrdU for at least two cell cycles.

### Validation of Rerep-Seq through simulated DNA rereplication in *S. cerevisiae*

**Figure 2.**
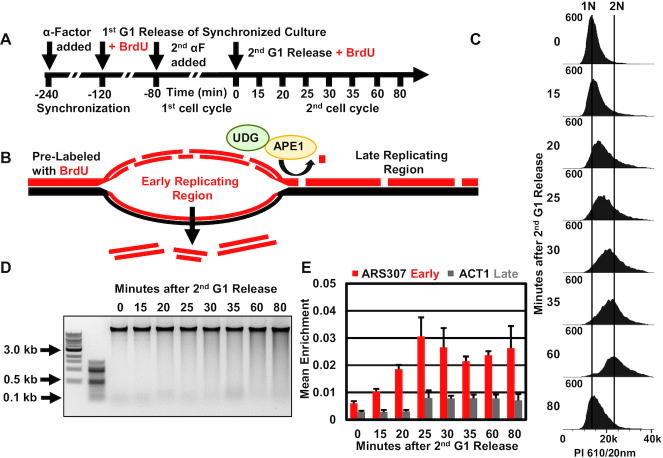
Simulation of DNA Rereplication in yeast cells. (**A**) Schematic of simulated DNA rereplication in yeast cells. Yeast synchronized with α-Factor then released into fresh media containing 0.1 mM BrdU followed by a second arrest and release in BrdU with samples collected at the indicated time points. (**B**) Schematic of selective fragmentation and enrichment of early replicating regions in the second cell cycle. After the first cell cycle, one DNA strand is labeled with BrdU (pre-labeled). As the synchronized culture starts the second S-phase, early replicating regions incorporate BrdU into both DNA strands and are now prone to fragmentation and enrichment. (**C**) Confirmation of cell cycle progression by flow cytometry. (**D**) Fragmentation of DNA increases as yeast progress through the second cell cycle. (**E**) Confirmation of enrichment of early replicating region. Equal volumes of gel extracted fragmented DNA analyzed by qPCR with primers against early (ARS307) and late (ACT1) replicating regions. Data represent mean enrichment from an average of three biological replicates (*n* = 3), error bars represent the SEM.

The lack of previous technology to evaluate rereplicated DNA presented a unique problem for validating Rerep-Seq using regions known to rereplicate. To overcome this issue, we simulated DNA rereplication by double labeling cells through a replication timing experiment in a manner that known early replicating regions would replicate a second time (mimicking rereplication) and act as proxies for DNA rereplication (Figure 2A and B). To validate this approach, we first arrested the culture in α-factor to synchronize the cells in G1 (Figure 2A and C). We then released the cells into media containing BrdU and after 40 min added α-factor again to synchronize the cells through one division at G1 (Figure 2C). We then released the cells a second time into media containing BrdU and collected DNA from cells at the indicated time intervals following release. Using this approach, during the second cell cycle, DNA should double strand BrdU label early replicating regions before late replicating regions (Figure 2B). In this second release, early replicating regions should preferentially enrich from Rerep-Seq first followed by detection of later replicating regions at later time points (Figure 2C and D). For comparison we also isolated DNA labeled completely in asynchronous culture for three cell cycles. As expected, we observed increased fragmentation as cells progressed through the second cell cycle (Figure 2D).

We analyzed the fragmented DNA prior to library preparation by qPCR. We utilized primers to a known early replicating region, a sequence defined DNA replication origin in yeast called Autonomous Replicating Sequence 307 (ARS307), and the late replicating region containing the gene ACT1. We determined that Rerep-Seq was able to specifically enrich early replicating regions prior to detection of later replicating regions (Figure 2E) (18). The verified samples were subjected to library preparation by Illumina's Nextera DNA Flex Library Prep Kit protocol, although standard adapter ligation and other library preparation procedures should work on the fragmented DNA. Importantly, including an *in vitro* DNA repair step (we utilized NEBs FFPE repair kit to fix nicks and oxidation) prior to starting the Nextera DNA Flex Library Prep protocol significantly increased library yields (data not shown) and we recommend including this repair step.

### Normalization of Rerep-Seq reads

In both yeast and human cells, mitochondria replicate asynchronously (regardless of cell-cycle arrest) and much faster than the genomic DNA (20,21). This means that, in practice, all the mitochondrial DNA is systemically double-labeled with BrdU and thus should be equally digested in each sample. We elected to use the mitochondrial DNA as an internal control for normalization. A BrdU labeled spike-in, such as a labeled plasmid, could also be used for normalization if desired, however the internal control of labeled mitochondrial DNA is preferable under most circumstances. After read normalization to RPM, individual samples were scaled based on the proportion of reads that aligned to the mitochondria (see ‘Materials and Methods’ section).

### Rerep-Seq enriches early replicating DNA

**Figure 3.**
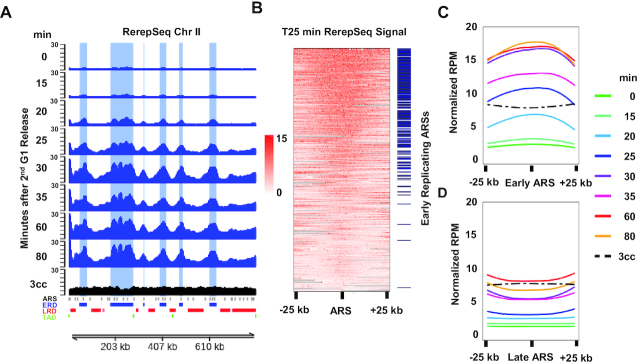
Global enrichment of early replicating regions in yeast using Rerep-Seq. (**A**) Rerep-Seq at Chr II as cells progress through S-Phase. RPM normalized, mitochondrial scaled Rerep-Seq signal. Tracks represent the average of three biological replicates for each time point (two for three cell cycle labeling, 3cc). Early time points exhibit enrichment at early firing ARS (ERDs, in blue; LRDs, in red at bottom of plot) followed by a broadening of signal into late replicating regions as cell progress through S-phase. ARSs are indicated by grey tick marks at bottom of plot. (**B**) Heat Map of Rerep-Seq surrounding 410 Confirmed ARSs at 25 min Post G1 Release. Centered are 410 confirmed ARSs ±25 kb, sorted by Rerep-Seq signal intensity. Blue ticks indicate early firing ARSs. (**C** and**D**) Meta analysis of enrichment at all ERDs (C) and all LRDs (D).

Rerep-Seq signal from our replication timing experiment was expected to exhibit peaks at early firing ARSs in early time points, followed by gradual broadening of peaks into late replicating regions with eventual fusion of peaks and flattening of signal as cells progressed through the second cell cycle. As expected, Rerep-Seq signal peaks emerge from early firing ARSs at 15 min post G1 release. These peaks increased in intensity, broaden and eventually merge with late replicating regions as cell progress through S-phase (Figure 3A). The pattern is clearly evident from each of three biological replicates (Supplementary Figure S1) and maintained when the replicates are averaged together (Figure 3A). Importantly, Rerep-Seq performed on cells labeled for more than three generations with BrdU does not demonstrate enrichment of early replication domains (Figure 3A, cc sample).

To further validate Rerep-Seq enrichment of early replicating regions, we classified 410 confirmed ARS elements as early or late replicating regions (17). We defined ERDs as regions in the top quartile of signal and LRDs the bottom quartile of signal in replication timing data reported previously (17–18,22–23). We then plotted the Rerep-Seq signal 25 min post-release within 25 kb of each ARS. The ARSs were sorted in order of the Rerep-Seq signal intensity from highest to lowest. We observed strong Rerep-Seq enrichment at early replicating ARSs indicated by blue tick marks (Figure 3B). A metanalysis of both early ARS and late ARS (Figure 3C and D) demonstrates that signal enriches over the early replicating ARS sequences in early time points following release, while later regions did not gain intensity until cells have progressed further through cell cycle. Together, these data demonstrate Rerep-Seq enriches double BrdU labeled DNA in a temporal and locus-specific manner.

### Validation of Rerep-Seq through simulated DNA rereplication in human cells

**Figure 4.**
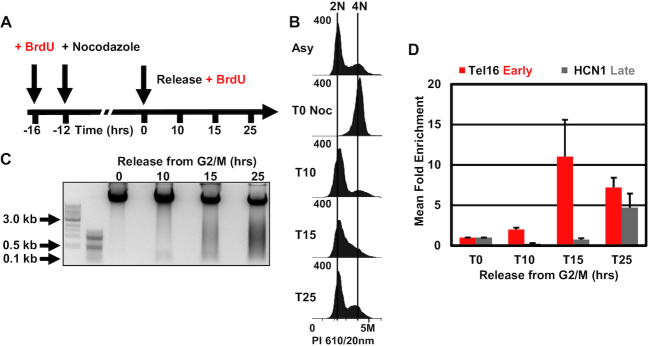
Simulation of DNA Rereplication in human cells. (**A**) Schematic of simulated DNA rereplication in human cells. MDA-MB-231 cells were treated with BrdU for 4 h followed by a 12-h treatment with nocodazole (with BrdU) to generate a synchronized culture arrested at G2/M with single strand BrdU incorporation. G2/M arrested cells were released into fresh media with BrdU. Samples were collected at 0, 10, 15 and 25 h for analysis by flow cytometry and Rerep-Seq. (**B**) Confirmation of cell-cycle progression by flow cytometry. (**C**) Fragmentation of DNA increases as cells progress through the second cell cycle. (**D**) Enrichment of early replicating human regions. Mean fold enrichment of qPCR signal over T0, using primers against the early replicating region on Chr16-telomere and the late replicating region containing the HCN1 gene. Data represent the average of three biological replicates (*n* = 3), error bars represent the SEM.

To validate Rerep-Seq in human cells, we performed a similar synchronized replication timing experiment. Although human cells do not have sequence defined origins, such as the yeast ARSs, DNA replication does follow an orchestrated timing program with defined early and late replicating regions (24). We labeled MDA-MB-231 cells for 4 h with BrdU prior to inducing a G2/M arrest with nocodazole for 12 h. This allowed one cell cycle of labeling with BrdU (Figure 4A). We collected G2/M arrested cells, washed them two times and released into fresh media containing BrdU. As cells entered the subsequent S phase, early replicating regions should acquire second strand BrdU labeling first, mimicking rereplication of these regions. (Figure 4A). We collected cells at 0, 10, 15 and 25 h post-release and analyzed their DNA content by flow cytometry to confirm cell cycle progression (Figure 4B). Purified genomic DNA from these cultures was subjected to the Rerep-Seq digest procedure (Figure 4C). Fragmented DNA, sizes ranging from 100 to 3000 bp, was separated on an agarose gel, excised, purified, repaired and then analyzed for early and late replicating regions by qPCR.

As expected, we observed an increase in DNA fragmentation as cells progressed through S-phase (Figure 4C). This correlated with Rerep-Seq signal enrichment of the early replicating sub-telomeric region of chromosome 16 at 10 and 15 h post-release, while the late replicating region at the HCN1 gene was not enriched until 25 h post-release (Figure 4D) (25). We then processed these validated samples for sequencing using Illumina Nextera DNA Flex Library Prep Kit.

**Figure 5.**
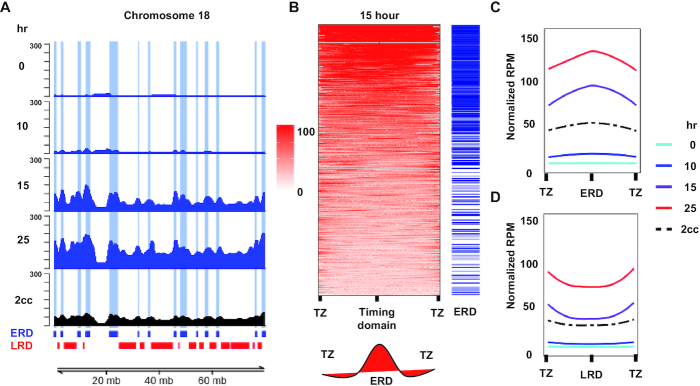
Global enrichment of early replicating regions in human cells using Rerep-Seq. (**A**) Rerep-Seq at Chr 16 as cells progress through S-Phase. RPM normalized and mitochondrial DNA scaled Rerep-Seq signal exhibits enrichment at ERDs (blue), followed by a broadening of signal into LRDs (red) as cell progress through S-phase. (**B**) Heat Map of Rerep-Seq Surrounding ERDs and LRDs 15 h post-nocodazole release. Heatmap is centered on either the ERD or LRD of interest and extends to the midpoint of the adjacent TZ. Each region was scaled from 0 to 100% to size normalize the ERDs and LRDs. The heatmap is sorted from highest to lowest based on signal intensity. Blue ticks indicate the ERDs. (**C** and **D**) Meta analysis of enrichment at all ERDs (**C**) and all LRDs (**D**).

For comparison to our Rerep-Seq data, we used publicly available replication timing data from the related MCF-7 breast cancer cell line (16,26). This replication timing data segmented the genome into ERDs, LRDs and TZs (16). Rerep-Seq signal from cells 10 to 15 h post-nocodazole release (correlating with early S phase; Figure 4B) strongly correlated with the predefined ERDs from MCF-7 cells (Figure 5A and Supplementary Figure S2). Twenty-five hours after release from nocodazole, late regions accumulated signal connecting the intervening ERDs.

To confirm ERD enrichment genome wide, we analyzed Rerep-Seq signal 15 h after release across 462 ERDs and 470 LRDs. To define each region, we included half the TZ on either side of the ERD or LRD. Since these domains ranged in size from 1000 to 20 272 173 bps, we scaled across each domain and plotted the data from 0% (middle of preceding TZ) to 100% (middle of following TZ). Each region was then sorted by signal intensity, which resulted in a clear enrichment of ERDs (blue ticks) with higher signal intensity than LRDs (Figure 5B). As with yeast, a meta-analysis of both ERDs and LRDs (Figure 5C and D) demonstrates that signal enriches over ERDs 10 and 15 h post-release, while LRDs gain intensity 25 h post-release. Importantly, MDA-MB-231 cells labeled for two complete cell cycles (2cc, Figure 5A, bottom track) did not exhibit enrichment of Rerep-Seq signal over ERDs or LRDs (Figure 5C and D).

### Analysis of rereplication following licensing deregulation in budding yeast

**Figure 6.**
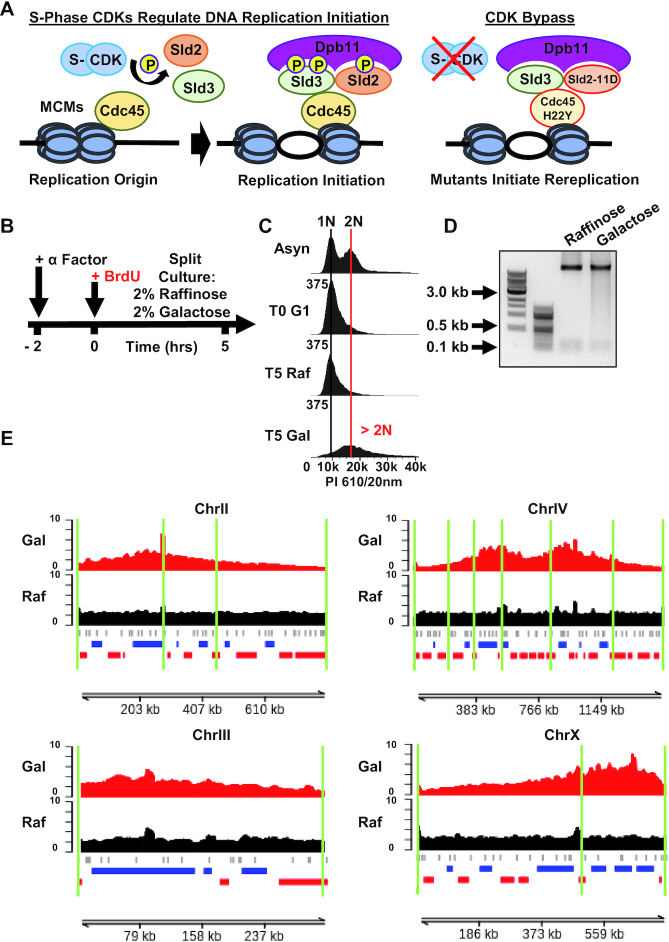
Deregulation of replication licensing induces non-random DNA rereplication in Yeast. (**A**) Schematic of genetic CDK bypass inducing DNA rereplication. Mutant Cdc45 H22Y (JET1) in combination with galactose inducible DBF4 and a galactose inducible phospho-mimetic Sld2-11D mutant, binds Sld3 and then facilitates the interaction between Sld3 and Dpb11 without phosphorylation. This results in activating DNA replication origins independently of S-phase CDK activity. (**B**) Schematic of CDK bypass experiment. YJB18 (JET1 GALp-sld2-11D, GALp-DBF4) cells grown in raffinose are then treated with α-factor to arrest culture in G1. Culture was then split into two: one grown in galactose the other in raffinose. BrdU was added to both. Cells were collected at 5 h post-galactose addition for DNA extraction and PI flow cytometry. (**C**) Cell-cycle analysis of YJB18 confirms increased DNA content indicative of DNA rereplication. (**D**) Fragmentation of rereplicated DNA by Rerep-Seq. (**E**) RPM normalized and mitochondrial DNA scaled reads were plotted. Data represent the average of three biological replicates. Gray ticks indicate ARS, ERDs are indicated in blue, LRDs in red. Green lines indicate position of Topologically Associating Domains (TADs).

Disruption of replication licensing has been shown to induce DNA rereplication (by flow cytometry) in yeast, drosophila and human cells (27–30). However, we know little about which sequences are rereplicated or if the whole genome is rereplicated evenly. To address this question, we took advantage of a CDK-bypass strain, courtesy of Dr Hiroyuki Araki, which harbors a mutant Cdc45 H22Y (JET1), that binds unphosphorylated Sld3, along with galactose inducible DBF4 and a phosphomimetic mutant of Sld2, sld2-11D. Together these mutants facilitate the interaction between Cdc45, Sld2, Sld3 and Dpb11, without phosphorylation, activating DNA replication origins independently of S-phase CDK activity (Figure 6A) (31). When grown in galactose, this strain exhibits greater than 2N DNA content even when arrested with α-factor, demonstrating bypass of normal licensing requirements (31). We transformed this strain to facilitate BrdU uptake with the BrdU-Inc cassette (15) to allow application of Rerep-Seq (Strain YJB18).

To determine which regions are rereplicated upon deregulation of licensing, we arrested cells in α-factor, shifted cells to galactose or raffinose and added BrdU for 5 h (Figure 6B). When YJB18 was grown in galactose, we observed the expected increase in cellular DNA content by flow cytometry (Figure 6C). YJB18 grown in raffinose was arrested in G1 using α-factor, then split into two cultures: one grown in raffinose with BrdU (no rereplication induction) and one grown in galactose with BrdU (induced rereplication). Five hours of galactose induction was sufficient for G1 arrested cells to attain greater than 2N DNA content and exhibit substantial DNA fragmentation using the Rerep-Seq digest protocol (Figure 6C and D). Fragmented DNA was gel purified, repaired then prepared for sequencing with Illumina Nextera DNA Flex Library Prep Kits.

Analysis of Rerep-Seq data demonstrated that not all chromosomes were rereplicated evenly (Figure 6E and Supplementary Figure S3). Generally, we observed large broad domains on multiple chromosomes. Some domains were centered on the chromosomes (chromosomes 12,16), some had two large domains (chromosomes 4,13), while other domains were enriched toward either end of the chromosome (chromosomes 2,3,5,7,10,11,14,15). Some chromosomes either did not rereplicate or were completely rereplicated that we could not distinguish using Rerep-Seq (chromosome 1,6,8,9). These broad domains spanned across multiple ARSs (Figure 6E and Supplementary Figure S3) making it difficult to determine the ARS of origin, if any. The broad domains spanned both early and late replication domains and contained both early and late firing ARS sequences. Consistent with crossing multiple replication timing domains, the rereplicated regions also crossed multiple topological boundaries defined previously in α-factor arrested cells (32). These data demonstrate that DNA rereplication following licensing disruption in yeast is non-random and does not result in uniform whole genome duplication.

## DISCUSSION

The prevailing view that cells replicate their DNA once, and only once, per cell cycle has recently been challenged by the implication of DNA rereplication into normal developmental programs, environmental stress response and role in cancer development and progression (29,33–34). Consistent with this idea, forced expression of DNA replication licensing factors can drive malignancy, and numerous replication factors are amplified or overexpressed in tumors (12,35–36). Further, forced rereplication of genes in yeast can drive gene amplification and aneuploidy (11). DNA rereplication has long been thought of as a source for gene amplification in tumors. However, there is little evidence in higher eukaryotes to support this model due to an inability to identify the sequences undergoing rereplication (37,38). As such, there is a clear need for new technology to identify which regions across the genome are prone to rereplication and under what conditions these amplifications are generated and persist.

Here we present Rerep-Seq to address this challenge, a technique that selectively fragments and enriches rereplicated DNA sequences in preparation for next generation sequencing. Of note, Rerep-Seq uses small amounts of DNA, works for any species or sample capable of incorporating BrdU with a rapid experimental timeline. These features will permit application of Rerep-Seq on diverse or limited samples such as small model organisms and cultured tissue biopsies. The ability to determine which genomic regions rereplicate under specific conditions will allow investigators to determine how rereplication is influenced by features such as sequence composition, presence of origins of replication, transcriptional regulators, enhancers of initiation, epigenetic marks and chromatin structure.

We validated Rerep-Seq by simulating DNA rereplication in yeast and human cells. Rerep-Seq was able to enrich ERDs when early regions should replicate in our validation experiments. We then used Rerep-Seq to identify regions of DNA that rereplicate in a yeast strain with deregulated licensing. We observed that DNA rereplication in these CDK bypass strains occurred in broad domains spanning multiple replication timing domains and crossing topological boundaries. These domains did not necessarily originate from early firing ARSs, as the domains contained both early and late replication domains, and some chromosomes containing early firing origins were not enriched at all. While we were unable to determine the reasons for why these specific domains rereplicated upon deregulation of origin licensing, their identification implicates specific licensing factors in limiting rereplication and their further study by Rerep-Seq may enable discovery of the underlying sequence, chromatin and biological functions of these domains. It will also allow comparison to rereplication induced through other pharmacological or genetic means to determine the commonalities that underlie DNA rereplication.

We anticipate Rerep-Seq will yield insight into the molecular mechanisms that generate rereplication dependent genomic plasticity and how such events contribute to development, evolution and cancer. Such knowledge has the potential to provide understanding of key developmental processes and how these processes go awry in disease.

## DATA AVAILABILITY

All sequencing data generated for this study has been deposited in the GEO database and is publicly available using the accession GSE143572. Human replication timing domains were taken from GSE53984. Yeast replication timing data was taken from GSE48212. Figures 3, 5, 6 and Supplementary Figures S1–3 have associated raw data deposited in the GEO submission.

## Supplementary Material

gkaa197_Supplemental_Files
