# Supplementary material for: Isolation and analysis of rereplicated DNA by Rerep-Seq
Source: Nucleic Acids Res. 2020 Apr 2;48(10):e58. doi: 10.1093/nar/gkaa197 (PMC7261181; doi:10.1093/nar/gkaa197)
Supplement: gkaa197_Supplemental_Files [file gkaa197_supplemental_files.zip › SupplementalFigureLegends.pdf]

Title: Isolation and analysis of rereplicated DNA by Rerep-Seq

Authors: Johannes Menzel[1,2], Philip Tatman[1,3], Joshua C. Black[1,2,3]*

Affiliations:
[1] University of Colorado Anschutz Medical Campus, Department of Pharmacology, 12800 E 19th Ave, Aurora CO 80045, USA
[2] University of Colorado Anschutz Medical Campus, Molecular Biology Graduate Program
[3] University of Colorado Anschutz Medical Campus, Medical Scientist Training Program

*Correspondence should be addressed to:

Joshua C. Black
Department of Pharmacology
University of Colorado School of Medicine Anschutz Medical Campus
Mail Stop 8303
12800 E. 19th Avenue Room P18-6116
Aurora, CO 80045, USA
Phone: 303-724-9991
Joshua.c.black@cuanschutz.edu

Supplemental Figure S1. Global enrichment of early replicating regions in yeast using Rerep-Seq**.** (**A**) Three replicates of Rerep-Seq at Chr II as cells progress through S-Phase. RPM normalized, mitochondrial scaled Rerep-Seq signal. Early time points exhibit enrichment at early firing Autonomously Replicating Sequences (ARSs) followed by a broadening of signal into late replicating regions as cell progress through S-phase. ARSs are indicated by grey tick marks, Early replicating domains (ERDs) in blue, late replicating domains (LRDs) in red, and boundaries of Topological Associated Domains (TADs) in green, at bottom of plot. Spearmen correlations between pairs of replicates are indicated to the right of the tracks for each time point. (**B**) GSEA analysis for enrichment of early replicating ARSs (left) and late replicating ARSs (right) at 25 minutes post release.

Supplemental Figure S2. Global enrichment of early replicating regions in human cells using Rerep-Seq. (**A**) Three replicates of Rerep-Seq at Chr 18 as cells progress through S-Phase. RPM normalized and mitochondrial DNA scaled Rerep-Seq signal exhibits enrichment at early replicating domains (ERDs, blue), followed by a broadening of signal into late replicating domains (LRDs, red) as cell progress through S-phase. Spearmen correlations between pairs of replicates are indicated to the right of the tracks for each time point. (**B**) GSEA analysis for enrichment of ERDs (left) and LRDs (right) at 15 hours post nocodazole release.

Supplemental Figure S3. Deregulation of replication licensing induces non-random DNA rereplication in budding yeast. (**A**) RPM normalized and mitochondrial DNA scaled reads were plotted for all chromosomes. Data represent the average of three biological replicates. Grey ticks indicate ARS, ERDs are indicated in blue, LRDs in red. Green lines indicate position of Topological Associated Domain (TAD) boundaries. (**B**) PCA of 3 replicates for both control (raffinose) and CDK bypass induction (galactose).
