# Supplementary figures and images for: Isolation and analysis of rereplicated DNA by Rerep-Seq
Source: Nucleic Acids Res. 2020 Apr 2;48(10):e58. doi: 10.1093/nar/gkaa197 (PMC7261181; doi:10.1093/nar/gkaa197)

Supplemental Figure S1

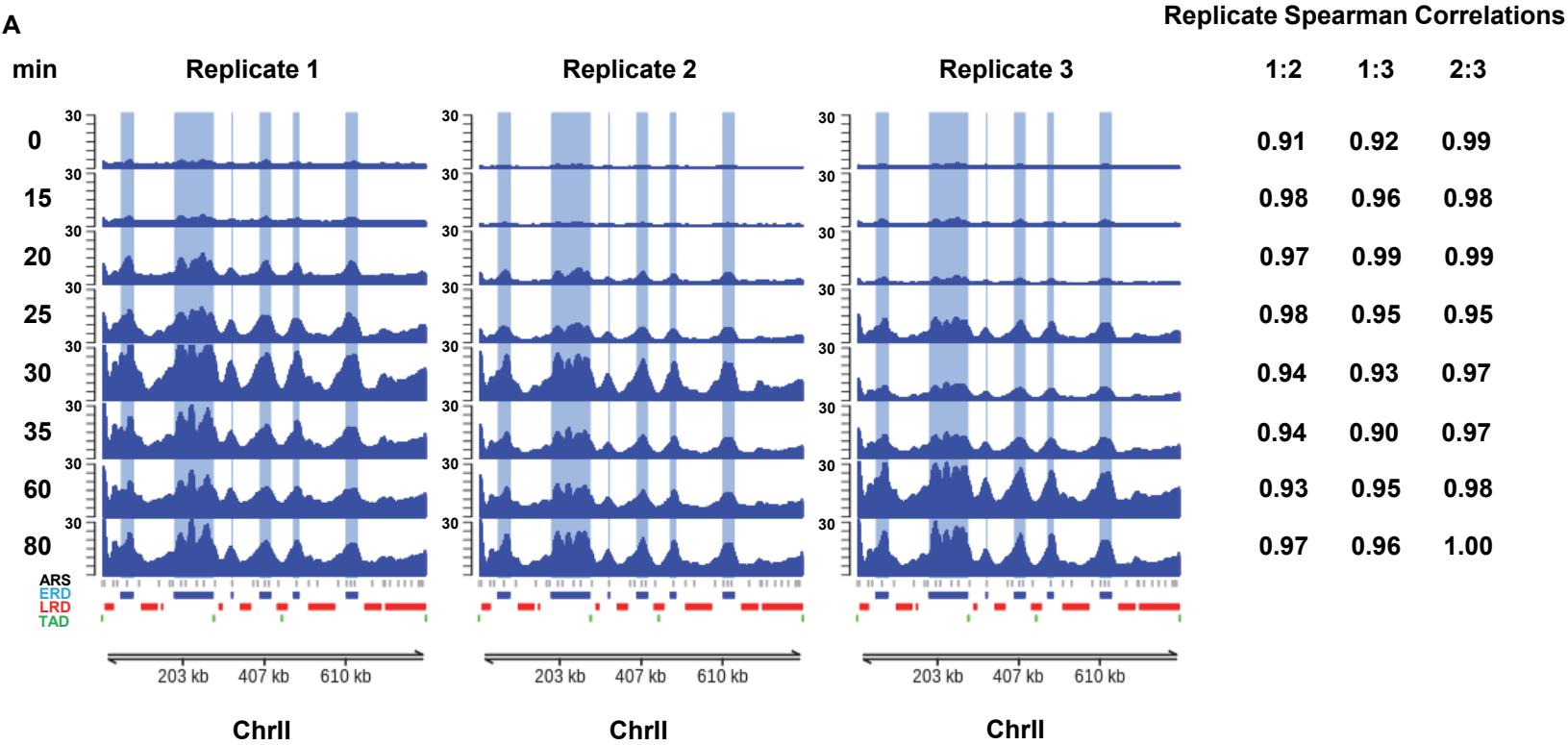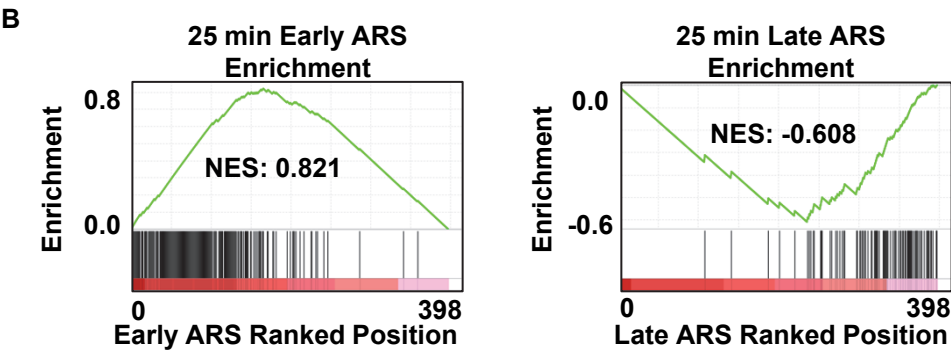

Supplemental Figure S2

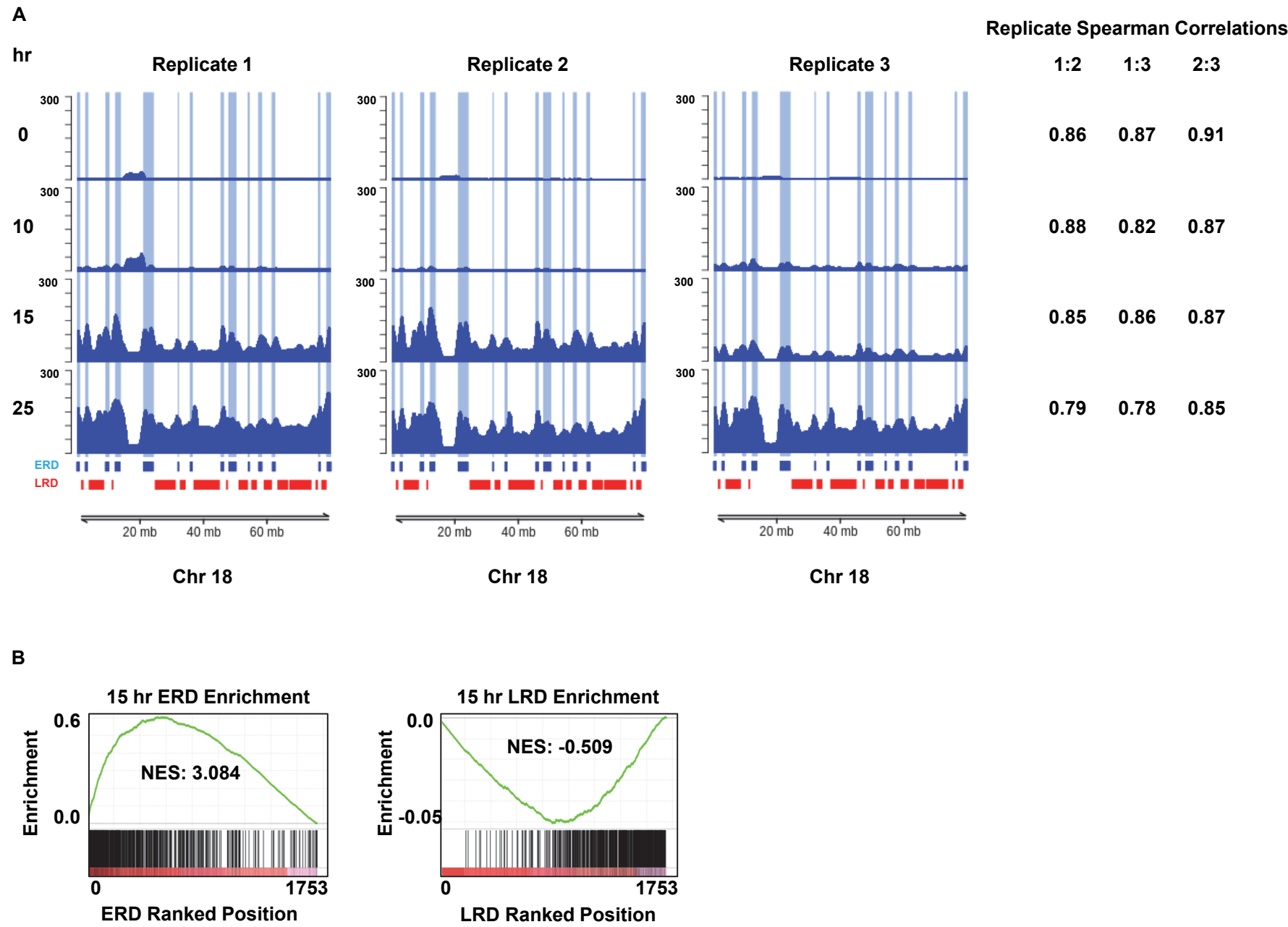

Supplemental Figure S3

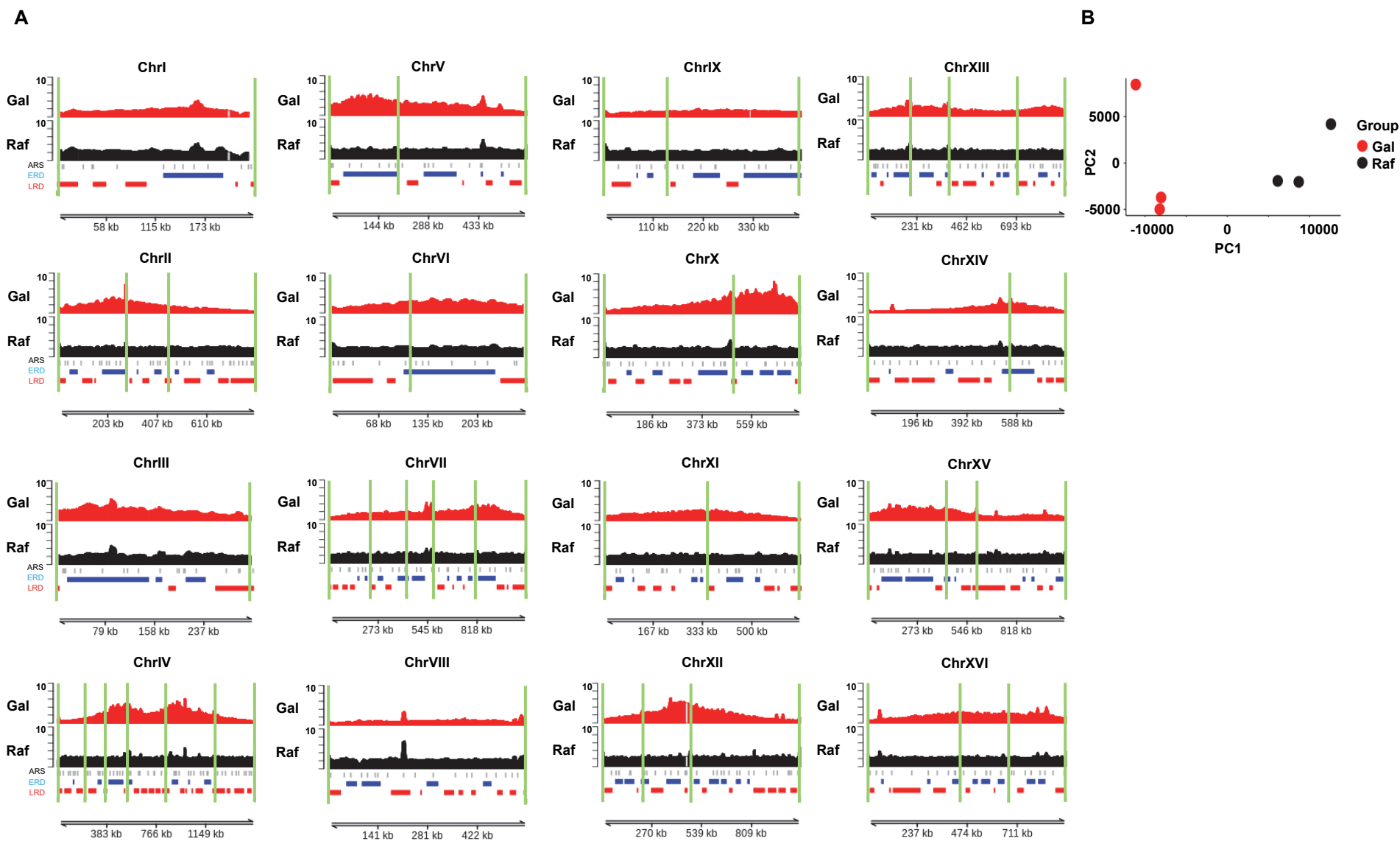

Supplement: gkaa197_Supplemental_Files [file gkaa197_supplemental_files.zip › MenzelSupplementalFigures.pdf]
